# Skeletal phenotypes in secreted frizzled-related protein 4 gene knockout mice mimic skeletal architectural abnormalities in subjects with Pyle’s disease from *SFRP4* mutations

**DOI:** 10.1038/s41413-022-00242-9

**Published:** 2023-02-20

**Authors:** Robert Brommage, Jeff Liu, David R. Powell

**Affiliations:** 1 Department of Metabolism Research, Lexicon Pharmaceuticals, The Woodlands, TX 77381 USA; 2 Present Address: BoneGenomics, The Woodlands, TX USA; 3 Present Address: Biogen, Cambridge, MA USA

**Keywords:** Bone, Bone quality and biomechanics

## Abstract

Mutations in *SFRP4* cause Pyle’s bone disease with wide metaphyses and increased skeletal fragility. The WNT signaling pathway plays important roles in determining skeletal architecture and SFRP4 is a secreted Frizzled decoy receptor that inhibits WNT signaling. Seven cohorts of male and female *Sfrp4* gene knockout mice, examined through 2 years of age, had a normal lifespan but showed cortical and trabecular bone phenotypes. Mimicking human Erlenmeyer flask deformities, bone cross-sectional areas were elevated 2-fold in the distal femur and proximal tibia but only 30% in femur and tibia shafts. Reduced cortical bone thickness was observed in the vertebral body, midshaft femur and distal tibia. Elevated trabecular bone mass and numbers were observed in the vertebral body, distal femur metaphysis and proximal tibia metaphysis. Midshaft femurs retained extensive trabecular bone through 2 years of age. Vertebral bodies had increased compressive strength, but femur shafts had reduced bending strength. Trabecular, but not cortical, bone parameters in heterozygous *Sfrp4* mice were modestly affected. Ovariectomy resulted in similar declines in both cortical and trabecular bone mass in wild-type and *Sfrp4* KO mice. SFRP4 is critical for metaphyseal bone modeling involved in determining bone width. *Sfrp4* KO mice show similar skeletal architecture and bone fragility deficits observed in patients with Pyle’s disease with *SFRP4* mutations.

## Introduction

Pyle’s disease, OMIM #265900, is an autosomal recessive familial metaphyseal dysplasia first identified in 1931^1–5^ with 51 subsequent reports describing 87 subjects from 21 countries. Radiographic abnormalities universally include Erlenmeyer flask deformities^6–8^ at the distal femur and humerus. Genu valgum of varying severity is present and the tibia has an S-shaped deformity at the midshaft. Cortical bone in limbs, clavicles, ribs and digits is exceptionally thin. Bone fractures, spine platyspondyly, and sclerosis of the skull base are often observed. A key observation is that height is normal, indicating there are no disturbances in longitudinal bone growth.

Mutations in the Secreted Frizzled-Related 4 gene (*SFRP4*) were shown to be responsible for Pyle’s disease in 2016^9^ and three independent confirmations have subsequently been reported.^10–12^ SFRP4 is a secreted WNT decoy receptor, structurally related to WNT Frizzled receptors, that binds WNTs to inhibit both canonical and non-canonical WNT signaling.^13–16^

Global *Sfrp4* gene disruption in Lexicon Pharmaceutical´s Genome 5000™ high-throughput phenotypic screen of 3 762 viable gene knockout (KO) mouse lines resulted in high trabecular bone mass but low cortical bone thickness.^17^ Additional *Sfrp4* KO mice were examined in a *Sfrp4/Wnt16* double KO study and the elevated trabecular bone mass with *Sfrp4* KO was not influenced by simultaneous *Wnt16* deletion. Midshaft femurs from these DKO mice had intermediate femur cortical cross-sectional area between elevated *Sfrp4* KO and reduced *Wnt16* KO values, indicating these two genes have independent actions on bone width. Bones from DKO mice had lower cortical thickness than the already reduced values in both single KOs but spontaneous fractures were not observed.

The first publication linking Pyle’s disease to human *SFRP4* mutations^9^ included Lexicon data showing *Sfrp4* KO mice have reduced midshaft femur cortical thickness from 4 weeks through 68 weeks of age and reduced femur shaft breaking strength at 24 weeks of age. We subsequently showed that *Sfrp4* KO mice have dramatically increased bone trabecularization in the femoral neck^18^ and mice with simultaneous KO of both *Sfrp4* and *Notum* have reduced cortical bone thickness, like values in *Sfrp4* KO mice rather than elevated values observed in *Notum* KO mice.^19^

Two independent laboratories also examined *Sfrp4* KO mice^9,20,21^ with agreement concerning greatly elevated trabecular bone mass, increased cortical bone width, and reduced cortical bone thickness. Here, we confirm and extend these observations to multiple ages and skeletal sites. *Sfrp4* KO mice have increased spine compressive breaking strength and normal ovariectomy-induced bone loss. Treating young adult *Sfrp4* KO mice with zoledronic acid, an inhibitor of osteoclastic bone resorption, does not influence normal gains in trabecular bone mass.

## Results

Lexicon’s high-throughput bone phenotyping campaign examining 3 762 viable gene KO mouse lines^9^ identified several skeletal abnormalities in *Sfrp4* KO mice. Body vBMD (Z score = minus 1.6) was reduced but spine LV5 trabecular bone volume (*Z* score = plus 2.6) and midshaft femur total area (*Z* score = plus 2.1) were elevated. Midshaft femur cortical thickness had the lowest value of all 3 343 genes examined. Figure S1 shows histograms with these data.

Further studies examined 7 cohorts of *Sfrp4* KO mice to determine the effects of age, sex, heterozygosity, ovariectomy, zoledronic acid treatment, and simultaneous KOs of *Wnt16* and *Notum* (Table S1). The numbers of *Sfrp4* mutant mice reaching weaning followed expected Mendelian inheritance ratios with 1 101 weanlings divided among 290 wildtype, 560 heterozygous and 251 homozygous mice (*P* = 0.21 by Chi-squared analysis). *Sfrp4* KO mice remained generally healthy with normal viability through 2 years of age (Fig. S2), but 6 of 14 KO mice (7 males and 7 females) displayed a hunchback with stiff thoracic spine when examined visually at 77 weeks of age. *Sfrp4* KO mice had normal body weight, fat content and lean body mass (Table S2). Apart from elevated platelet levels in female mice, clinical chemistry and blood cell count values were normal in *Sfrp4* KO mice at 2 years of age (Tables S3, S4).

**Fig. 1:**
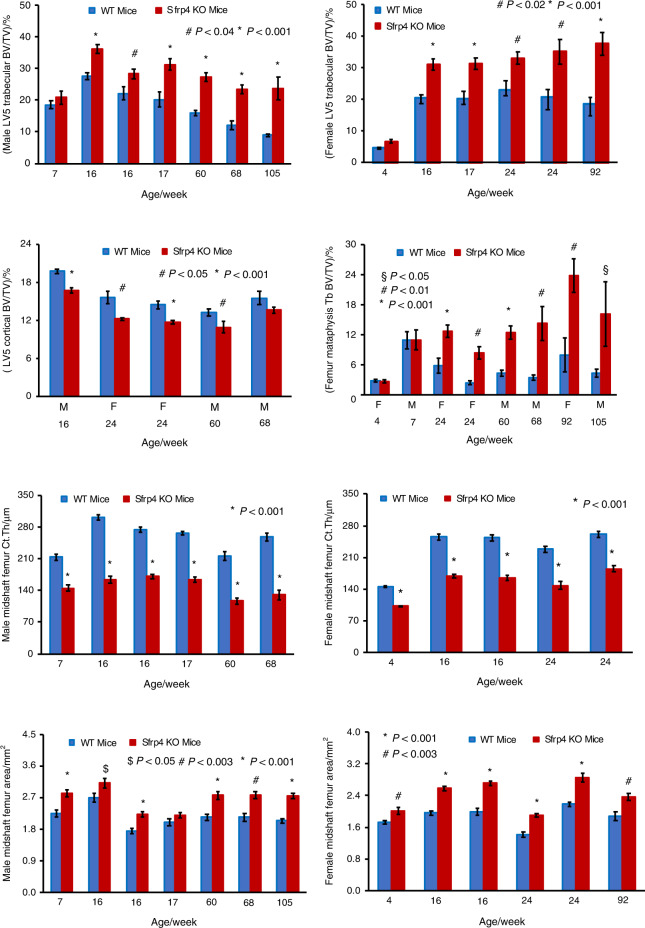
Age-dependence of skeletal phenotypes in Sfrp4 KO mice. All data are means ± SEMs for 7 to 14 mice. Statistical analyses by student’s *t*-test

**Fig. 2:**
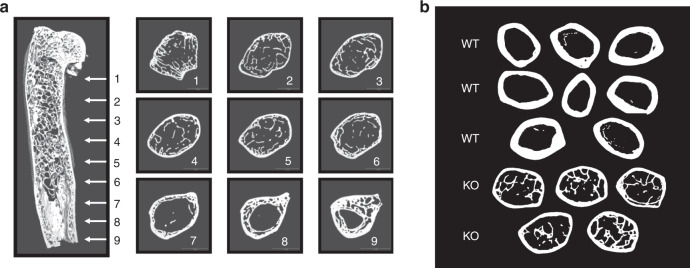
Femur microCT images obtained from a male mouse at 105 weeks of age. **a** Longitudinal image from one *Sfrp4* KO femur. **b** Midshaft cross-section images of 8 WT and 5 KO femurs

The following phenotypes were consistently observed in multiple cohorts: (1) elevated trabecular bone mass (BV/TV) in LV5 and distal femoral metaphysis (Fig. 1), (2) elevated LV5 trabecular number (Fig. S3), (3) elevated midshaft femur cross-sectional area (Fig. 1), (4) reduced midshaft femur cortical thickness (Fig. 1), and (5) reduced midshaft femur mBMD and vBMD measured by microCT (Fig. S4). LV5 trabecular thickness was normal (data not shown). Femur lengths, measured at 17, 24, and 60 weeks of age were within 2% of control values (Table S5), indicating these skeletal phenotypes did not result from alternations in longitudinal bone growth. Although DXA body BMD values were minimally reduced with *Sfrp4* KO, spine BMD was generally elevated and femur BMD was generally reduced (Table S6).

Male *Sfrp4* heterozygous and KO mice were examined at 60 weeks of age (Table S7). In addition to the parameters described above, KO mice had increased metaphyseal trabecular numbers in the distal femur and proximal tibia. Metaphyseal trabecular thickness was reduced at both skeletal sites. Consistent with phenotypes observed in the midshaft femur, tibial cortical bone (at the tibia-fibula junction) had elevated total area (mineralized bone plus marrow) and reduced cortical thickness. Metaphyseal cross-sectional areas were elevated 2-fold in both distal femur and proximal tibia. *Sfrp4* heterozygous mice had elevated LV5 trabecular BV/TV, elevated metaphyseal trabecular numbers in both distal femur and proximal tibia and reduced trabecular thickness in the proximal tibia metaphysis. All other bone parameters were normal in *Sfrp4* heterozygous mice.

A longitudinal microCT slice image of a representative male *Sfrp4* KO femur at 2 years of age shows extensive marrow cavity trabecularization (Fig. 2). Representative midshaft femur cross-section images are also shown in Fig. 2, with multiple trabeculae present in bones from all 5 *Sfrp4* KO mice but few or no marrow cavity trabeculae for the 8 wild-type (WT) mice. Histological observations (data not shown) from plastic-embedded sections of midshaft femurs of these mice showed no fluorochrome labels, indicating the absence of ongoing periosteal bone formation in wild-type (WT) or KO mice. Endocortical surfaces of bones from WT mice were undergoing minimal focal remodeling.

**Fig. 3:**
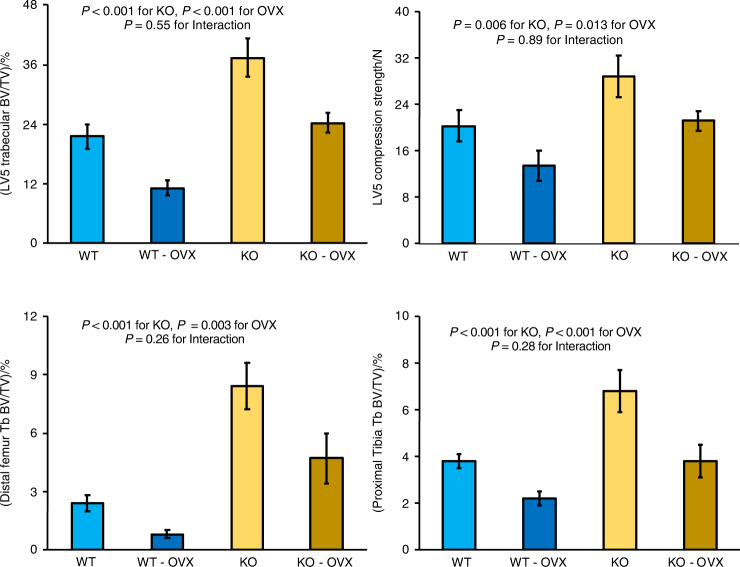
Effects of Sfpr4 KO and ovariectomy on trabecular bone parameters. Mice underwent ovariectomy or sham surgery at 16 weeks of age and bones were analyzed at 24 weeks of age. Data are means ± SEM for 9 to 11 mice per group, with statistical analysis by two-factor ANOVA

In an 8-week ovariectomy study (Fig. 3) KO of *Sfrp4* in sham-OVX mice led to increased trabecular bone volume in the LV5 vertebral body (74%), distal femur metaphysis (3.5-fold) and proximal tibia metaphysis (3.1-fold). In WT mice, ovariectomy resulted in loss of trabecular bone in the LV5 vertebral body (48%), distal femur metaphysis (66%) and proximal tibia metaphysis (44%). In *Sfrp4* KO mice, ovariectomy led to similar fractions of trabecular bone loss, being 65% in the LV5 vertebral body, 44% in the distal femur metaphysis and 44% in the proximal tibia metaphysis. Compression strength testing of the LV5 vertebral body demonstrated the expected effects of ovariectomy and *Sfrp4* KO. Maximum load of the vertebral body was increased by 43% in *Sfrp4* KO mice and decreased by 34% and 27% following ovariectomy in WT and *Sfrp4* KO mice, respectively. Compared to WT control values, trabecular bone following OVX in *Sfrp4* KO mice was similar in the proximal tibia, 13% higher in LV5 and 2-fold higher in the distal femur. Compression strength of LV5 vertebral body (including contributions from the cortical shell) was similar in sham-WT and OVX-*Sfrp*4 KO mice.

**Fig. 4:**
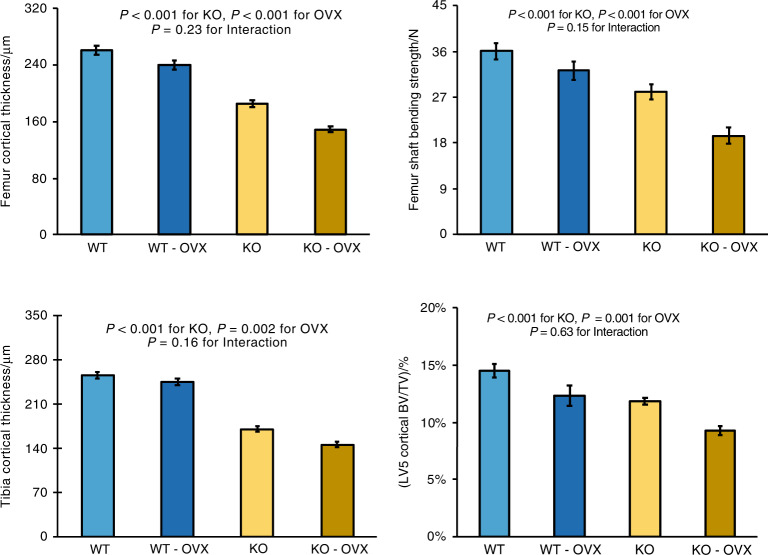
Effects of Sfpr4 KO and ovariectomy on cortical bone parameters. Mice underwent ovariectomy or sham surgery at 16 weeks of age and bones were analyzed at 24 weeks of age. Data are means ± SEM for 9 to 11 mice per group, with statistical analysis by two-factor ANOVA

OVX-induced loss of cortical bone occurred in the midshaft femur, midshaft tibia and LV5 vertebral body for both control and *Sfrp4* KO mice (Fig. 4). Femoral cortical thickness losses were 8% (21 µm) in WT and 19% (36 µm) in *Sfrp4* KO mice. Similarly, tibial cortical thickness losses were 4% (10 µm) in WT and 14% (24 µm) in *Sfrp4* KO mice. For all four groups, midshaft femur and tibia cortical thickness values were highly correlated (Fig. S5). LV5 cortical BV/TV losses were 15% in WT and 21% in *Sfrp4* KO mice. OVX-induced reductions in midshaft femur breaking strength were 11% (3.9 N) in WT and 31% (8.6 N) in KO mice. Compared to WT control values, the combination of OVX and *Sfrp4* KO reduced LV5 cortical BV/TV by 36%, femoral cortical thickness by 43%, tibial cortical thickness by 40% and femoral strength by 46%. Femoral 4-point bending strength across all bones (Fig. S6) was correlated to cortical thickness (R^2^ = 0.70) but neither total area (R^2^ = 0.27), nor polar moment of inertia (R^2^ = 0.08).

Male and female WT and *Sfrp4* KO mice were treated with the bisphosphonate zoledronic acid at 8 weeks of age. At this age *Sfrp4* KO mice have reduced cortical bone thickness, but normal trabecular bone mass (Fig. 1). Male and female mice were treated for 60 and 16 weeks, respectively. For male mice, analyses of serum levels of PINP and CTX at 50 weeks of age to assess bone turnover (remodeling) showed no effect of *Sfrp4* KO but 40% reductions with zoledronic aid treatment (Table S8).

**Fig. 5:**
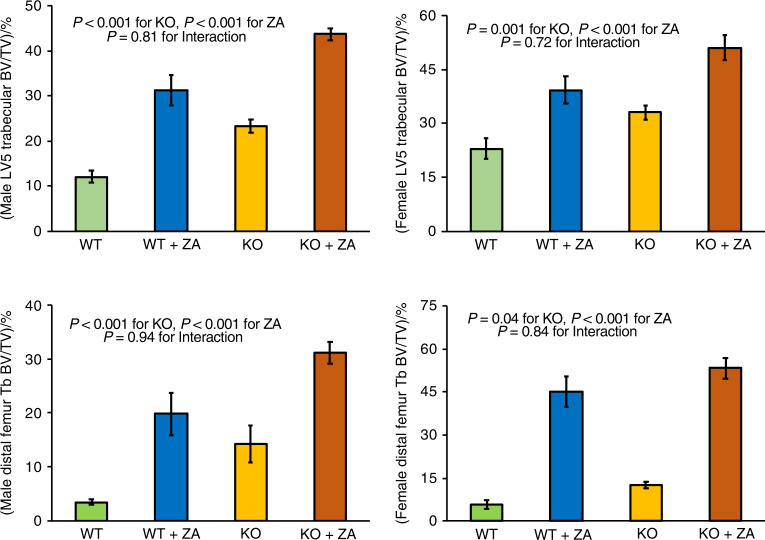
Effects of Sfpr4 KO and zoledronic acid (ZA) treatment on trabecular bone parameters. Mice were dosed intraperitoneally with saline or zoledronic acid (20 μg·kg^−1^) at 8 weeks of age. Male and female mice were examined at 68 and 24 weeks of age, respectively. Data are means ± SEM for 5 to 8 mice per group with statistical analysis by two-factor ANOVA

Both male and female *Sfrp4* KO mice had elevated trabecular bone mass in both LV5 vertebral body and the distal femur metaphysis, with zoledronic acid treatment resulting in further additive increases in trabecular bone mass (Fig. 5). These findings demonstrate that zoledronic acid treatment does not interfere with the development of elevated trabecular bone mass with *Sfrp4* disruption. Zoledronic acid treatment did not differentially affect midshaft femur cortical bone width or thickness, nor LV5 cortical bone mass, in WT or KO mice (Fig. S7). These findings suggest simultaneous treatment with a SFRP4 neutralizing antibody and a bisphosphonate in adult osteoporosis patients might promote trabecular bone gain while inhibiting further cortical bone loss.

Analysis of published gene expression databases showed that *Sfrp4* expression was stimulated 7-fold during BMP2-induced differentiation of murine C2C12 myoblasts into osteoblasts (Fig. S8).

## Discussion

**Fig. 6:**
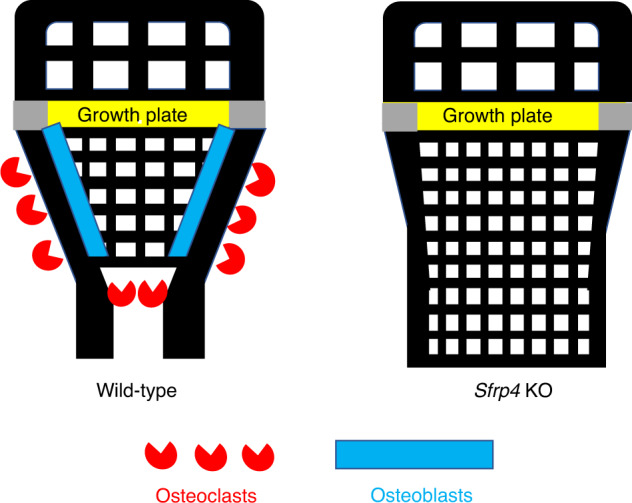
Model of SFRP4 Actions on Metaphyseal Bone Development. Mineralized bone isshown in black; the growth plate is shown in yellow; and gray regions indicate theperichondrium. Wide metaphyses (Erlenmeyer flask deformity) result from reduced boneresorption by periosteal osteoclasts. Reduced endocortical bone formation contributes to lowcortical bone thickness. We propose the abnormal persistence of diaphyseal trabeculae results from failure of osteoclastic resorption of these trabeculae near the metaphyseal - diaphyseal junction. Bone length is unaffected, indicating normal endochondral growth plate activities

Pyle’s disease is a developmental familial skeletal dysplasia resulting from disturbances in metaphyseal bone modeling (Fig. 6), a complex process involving periosteal bone resorption, endocortical bone formation and coalescence of calcified cartilage trabeculae into cortical bone.^22–26^ Serial wrist radiographs obtained during healthy adolescence showed distal radius rates of periosteal resorption and endocortical apposition averaged 8 μm·d^−1^ and 10 μm·d^−1^, respectively.^27^ Although human short stature can result from disruptions in at least 60 genes influencing longitudinal bone growth,^28^ bone lengths are unaffected in Pyle´s disease subjects and *Sfrp4* KO mice. Therefore, SFRP4 does not appear to influence formation and maintenance of articular cartilage and epiphyseal bone, nor growth plate dynamics.

Failure of normal metaphyseal bone modeling results in radiographic Erlenmeyer flask deformities,^6^ also known as metaphyseal widening, clubbing, flaring and undermodeling. Besides Pyle’s disease, human skeletal dysplasias with normal bone lengths and Erlenmeyer flask deformities occur with mutations in *ANKH* (craniometaphyseal dysplasia,^29^ *COL10A1* (Schmid metaphyseal dysplasia,^30,31^ *FLNA* (frontometaphyseal dysplasia,^32^ *GBA* (Gaucher’s disease,^33^ *LRRK1* (osteosclerotic metaphyseal dysplasia,^34^ *PTH1R* (Jansen’s metaphyseal chondrodysplasia,^35^ and CLCN7,^36,37^ *PLEKHM1,*^38^ and *SNX10*^39^ each resulting in osteopetrosis. COL10A1 is a cartilage structural component; PTH1R and SFRP4 are involved in cell signaling pathways; with ANKH, CLCN7, FLNA, GBA, LRRK1, PLEKHM1 and SNX10 all having roles in bone resorbing osteoclasts. Osteoclast dysfunction contributes to failure of metaphyseal periosteal bone resorption leading to the Erlenmeyer flask deformity.

There are no apparent bone architecture differences between male and female Pyle’s disease subjects nor *Sfrp4* KO mice. Bone dysplasias in Pyle’s disease subjects and *Sfrp4* KO mice appear during growth and are not progressive in adults. SFRP4 is unlikely to be involved in postmenopausal osteoporosis as cortical and trabecular bone losses following ovariectomy in adult mice was unaffected by *Sfrp4* KO. These observations suggest that inhibition of SFRP4, for example with a therapeutic neutralizing antibody, would not be a viable therapy for postmenopausal osteoporosis.

Standard clinical radiographs obtained in living Pyle’s disease subjects cannot show trabecular bone abnormalities occurring within bones. Iliac crest biopsies and 3-dimensional bone CT scans have not been performed to provide insights into trabecular bone architecture. Fortunately, Ingalls obtained a femur and tibia from a cadaver, first described in 1910,^40^ having classic Erlenmeyer flask deformities. He sectioned these bones longitudinally and published his descriptions in 1933 with a photograph of the sectioned bones.^41^ Extensive trabecular bone was observed within the distal three-fifths of this femur and proximal three-fifths of this tibia. Femoral Erlenmeyer flask enlargement was greater in the coronal than the sagittal plane.^42^ Similar observations were subsequently made on a human femur and tibia recovered from an archeological site in Peru.^43^ These bone phenotypes are consistent with *SFRP4* mutations but other genetic metaphyseal dysplasias are possible.

*Sfrp4* KO mouse limbs exhibit Erlenmeyer flask deformities observed in Pyle’s disease subjects. Metaphyseal cross-sectional areas are elevated 2-fold in the mouse distal femur and proximal tibia (Table S7) but by only 30% in the midshaft femur (Fig. 1) and tibia shaft at the fibula junction (Table S7). These observations are consistent with the excessively wide proximal tibiae and distal femurs previously observed in *Sfrp4 KO* mice.^9,20^

Mouse clavicles, ribs, digits, and the cranial base, sites with radiographic abnormalities in Pyle’s subjects, have not been examined as all mouse studies were performed prior to the 2016 identification of *SFRP4* mutations being responsible for Pyle’s disease.^9^ During development growth plates (synchondroses) separate the sphenoid, ethmoidal and basioccipital bones at the base of the skull.^44^ Presumably, disturbances in metaphyseal bone modeling similar to those observed in limbs is responsible for cranial base sclerosis observed in Pyle’s disease subjects. Skull shape appears normal as SFRP4 does not affect longitudinal growth. The femoral neck growth plate is located just below the femoral head and femoral necks in WT mice consist of predominately cortical bone but are extensively trabecularized in *Sfrp4* KO mice.^18^

Consistent with the autosomal recessive inheritance of Pyle’s disease, except for modestly elevated trabecular bone mass in spine, femur, and tibia, we did not observe skeletal phenotypes in heterozygous *Sfrp4* mice. Studies employing pQCT would presumably show excess trabecular bone within limbs of both subjects and possibly their heterozygous relatives.

The lifelong persistence of extensive trabecular bone networks within limb diaphyses from two human adult cadavers presumed to have Pyle’s disease^40,42^ and aged *Sfrp4* KO mice is unusual. We suggest that the normal resorption of entire trabeculae at the metaphyseal-diaphyseal junction does not occur in the absence of SFRP4, resulting in marrow cavity trabecularization within the diaphysis. Extensive trabecularization of the diaphyseal marrow cavity also occurs in adult mice with genetic disruptions of *Kindlin3*,^45^ *Lrrk1*^46^, *Schnurri3*,^47^ *Sgpl1*,^17,48^ *Slc37a2*^49^, and *Vhl*.^50^ Consistent with our suggestion, diaphyseal trabeculae in adult *Lrrk1* and *Vhl* KO mice contain remnants of calcified cartilage, presumably originating from the metaphyseal growth plate region. The extent of trabecular bone remodeling in adults is unclear. Levels of serum bone turnover markers PINP and CTX are normal in *Sfrp4* KO mice at 50 weeks of age, adult *Vhl* KO mice^50^ and in the few adult Pyle’s disease subjects examined.^9^

Normal gains in trabecular bone mass in both the distal femur metaphysis and LV5 vertebral body after 8 weeks of age in *Sfrp4* KO mice following treatment with zoledronic acid, an osteoclast inhibitor, are consistent with elevated trabecular bone formation in *Sfrp4* KO mice observed by others.^9^ Endocortical and trabecular bone loss in femurs, tibias and vertebral bodies following ovariectomy were unaffected by *Sfrp4* gene disruption. These results suggest SFRP4 specifically inhibits metaphyseal endocortical bone resorption during longitudinal growth.

Metaphyseal bone dysmorphology observed with *SFRP4* disruption is consistent with the expression patterns of *Sfrp4* in mouse bone. Robust *Sfrp4* expression in embryonic^51^ and neonatal^52^ mouse femoral metaphysis is observed by in situ mRNA hybridization. Similarly, robust skeletal *Sfrp4* expression, viewed in whole-mount mouse bones after LacZ-driven X-Gal staining, occurs in embryonic mouse midshaft femur and the femur metaphysis at 3 weeks^20^ and 12 weeks of age.^53^ *Sfrp4* expression in adult mouse bone lining cells was identified by RT-PCR after FACS isolation.^54^ *SFRP4* is highly expressed in primary cultures of human osteoblasts.^55^ *Sfrp4* expression is stimulated 5-fold following PTH treatment of osteocyte Ocy454 cells^56^ and, as described above, 7-fold during BMP2-induced differentiation of murine C2C12 myoblasts into osteoblasts.^57^ These studies all suggest *Sfrp4* is expressed by bone cells. Overexpression of *Sfrp4* in mice reduces trabecular bone mass.^52,58^

Results from the present study, our previously published data,^17–19^ and findings by two independent laboratories^9,20,21^ all support *Sfrp4* KO mice are a good model for human Pyle’s disease resulting from human *SFRP4* mutations. Bone dysplasias, including Erlenmeyer flask deformities, occur during growth and persist in adults. Human height and mouse bone length are normal. Partially penetrant hunchback posture occurs in adult mice and platyspondyly has been reported in humans.^8^ There are extensive trabecular bone networks within adult diaphyseal marrow cavities and cortical bone thickness is reduced. Mouse longevity and serum chemistry values are both unaffected. Human fragility fractures are common and we previously reported^17^ that mouse femur shafts have reduced 4-point bending strength.

## Methods

Knockout mice were generated by homologous recombination techniques with disruption of the first exon of the *Sfrp4* gene (Fig. S9). All studies examined F2 hybrid littermate/cagemate mice derived from C57BL/6 J and 129SvEv parental strains generated and studied within the Lexicon vivarium.^19^ Mice were group-housed in micro-isolator cages within a barrier facility at 24 °C on a fixed 12-h light and 12-h dark cycle and fed Purina rodent chow No. 5001 (Purina, St. Louis, MO). The high-fat diet (45 kcal percent fat) employed in the primary screen was D12451i from Research Diets (New Brunswick, NJ USA). Diets and acidified water were provided ad libitum. All procedures involving use of live mice were conducted in conformance with Lexicon’s Institutional Animal Care and Use Committee guidelines that were in compliance with the state and federal laws and the standards outlined in the Guide for the Care and Use of Laboratory Animals (National Research Council, 1996). Quarterly sentinel surveillance showed no evidence of pathogenic rodent viruses, *Mycoplasma*, or *Helicobacter* species in the source colonies. All mice and samples were analyzed after investigator blinding to genotypes and experimental treatments.

Spine BMD and body composition were measured under isoflurane anesthesia using a PIXImus DXA (InsideOutside Sales, Fitchburg, WI) by standard techniques. Bone architecture was determined by microCT using a µCT40 (Scanco Medical, Brüttisellen, Switzerland) scanner employing a threshold of 240, an X-ray tube voltage of 55 keV, a current of 145 microamperes and an integration time of 200 microseconds. For cortical bone analyses in the femur (midshaft) and tibia (tibia-fibula junction), twenty slices were analyzed and total area of mineralized bone plus marrow was determined as an estimate of bone cross-sectional area. For LV5, the vertebral body was analyzed for trabecular bone and, in four cohorts, also for cortical bone. Trabecular bone analyses included the region inside the cortical shell. When cortical analyses were performed, the scans were first analyzed for trabecular bone and then a second contour was traced to include the cortical region of the (Fig. S10).

Bilateral ovariectomy or sham surgery was performed at 16 weeks of age, with bones examined 8 weeks later. Mice were administered 20 µg·kg^−1^ single doses of zoledronic acid intraperitoneally at 8 weeks of age, with bones examined at 24 weeks for females and 68 weeks for males. Zoledronic acid (*Zometa*, Novartis, Basel, Switzerland) was purchased from a pharmacy and diluted with sterile saline before use.

For male mice examined at 105 weeks of age, subcutaneous injections of sterile solutions of calcein (10 mg·kg^−1^) and demeclocycline (30 mg·kg^−1^) were given 26 and 5 days, respectively, prior to necropsy to label bone forming surfaces. Both calcein and demeclocycline were purchased from SigmaAldrich (St. Louis, MO USA). Femur shafts were embedded in methacrylate, sectioned at ~80 µm with a SP1600 Saw Microtome (Leica Biosystems, Buffalo Grove, IL USA) and visually examined by fluorescent microscopy.

For the ovariectomy study, LV5 vertebral body ultimate compression strength and femur shaft 4-point ultimate breaking strength were measured using an Instron 5500 Testing Unit at Numira Biosciences (Salt Lake City, UT USA). LV5 vertebral bodies were compressed at a rate of 0.6 mm·min^−1^. Femurs were mounted with a 2.5 mm inner support and a 7.0 mm outer support and 4-point loading was applied at a displacement rate of 3 mm·min^−1^.

The EMBL-EBI Expression Atlas^59^ was employed to search for relevant published mouse *Sfrp4* gene expression data. This search identified a dataset providing expression data (GeneChip™ Mouse Genome 430 2.0 Array for 45 000 probes) during BMP2-induced differentiation of murine C2C12 myoblasts into osteoblasts.^57^

Statistical evaluations were by Student’s *t*-test, one-factor ANOVA (with Dunnett’s test for multiple comparisons) or two-factor ANOVA, as appropriate. Sample numbers were determined from power calculations using values from Lexicon’s extensive database of skeletal phenotypes in KO mice.^17^

## Supplementary information

SFRP4 Manuscript Supplemental Material

## Data Availability

The datasets used and/or analyzed during the current study are available from the corresponding author on reasonable request.
