# Supplementary material for: Skeletal phenotypes in secreted frizzled-related protein 4 gene knockout mice mimic skeletal architectural abnormalities in subjects with Pyle’s disease from SFRP4 mutations
Source: Bone Res. 2023 Feb 20;11:9. doi: 10.1038/s41413-022-00242-9 (PMC9941579; doi:10.1038/s41413-022-00242-9)
Supplement: Supplementary file 1 — SFRP4 Manuscript Supplemental Material [file 41413_2022_242_MOESM1_ESM.docx]

Skeletal Phenotypes in Secreted Frizzled-Related Protein 4 Gene Knockout Mice Mimic Skeletal Architectural Abnormalities in Subjects with Pyle’s Disease from *SFRP4* Mutations

**Supplementary Information**

Figure S1: Identification of *Sfrp4* KO Mouse Skeletal Phenotypes during Lexicon’s Primary High-Throughput Screens

Figure S2: *Sfrp4* KO Mouse Viability Through 2 Years of Age

Figure S3: Elevated Vertebral Body Trabecular Number in *Sfrp4* KO Mice

Figure S4: Reduced Midshaft Femur mBMD and vBMD in *Sfrp4* KO Mice

Figure S5: High Correlation of Midshaft Femur and Midshaft Tibia Cortical Thickness in the Ovariectomy Study

Figure S6: Correlations of Femur 4-Point Bending Strength with Cortical Thickness and Total Area in the OVX Study

Figure S7: Effects of *Sfpr4* KO and Zoledronic Acid (ZA) Treatment on Cortical Bone Parameters

Figure S8: Upregulation of *Sfrp4* Expression in during BMP2 Induced Differentiation of Murine C2C12 Myoblasts into Osteoblasts

Figure S9: *Sfrp4* Gene Knockout Targeting Strategy and Southern Blot Analysis

Figure S10: Visualization of Spine LV5 Vertebral Body Cortical and Trabecular Bone Measurements

Table S1: *Sfrp4* KO Mouse Cohorts

Table S2: Body Composition and BMD Values in *Sfrp4* KO Mice

Table S3: Clinical Chemistry Data for *Sfrp4* KO Mice at 2 Years of Age

Table S4: Complete Blood Count Data for *Sfrp4* KO Mice at 2 Years of Age

Table S5: Femur Lengths of *Sfrp4* KO Mice

Table S6: Body, Spine and Femur DXA BMD Data

Table S7: Skeletal Parameters in Heterozygous and Homozygous *Sfrp4* KO Mice

Table S8: Serum levels of PINP and CTX in the Zoledronic Acid StudySupplemental Figure 1: Identification of *Sfrp4* KO Mouse Skeletal Phenotypes during Lexicon’s Primary High-Throughput Screens

Figures for body vBMD, LV5 BV/TV and femur cortical thickness have been presented previously (Brommage et al., 2014). Volumetric BMD (vBMD) is defined as BMD divided by bone area, employed to account for differences in bone size. The femur cortical thickness histogram does not include data for 23 KO lines examined on a pure C57BL/6 strain background, for which mean cortical thickness was 191 µm.

Brommage R, Liu J, Hansen GM, Kirkpatrick LL, Potter DG, Sands AT, Zambrowicz B, Powell DR, Vogel P. High-throughput screening of mouse gene knockouts identifies established and novel skeletal phenotypes. Bone Res. 2014; 2:14034.

Supplemental Figure 2: *Sfrp4* KO Mouse Viability Through 2 Years of Age

Cohort 4 mice were segregated by sex, with WT and KO mice housed together and initially 4 mice per cage. DEXA scans were performed at 52 and 78 weeks of age.

.

Supplemental Figure 3: Increased LV5 Trabecular Number in *Sfrp4* KO Mice

Supplemental Figure 4: Reduced Midshaft Femur mBMD and vBMD in *Sfrp4* KO Mice

Material BMD (mBMD) is defined as BMC/Mineralized Bone Volume

Volumetric BMD (vBMD) is defined as BMC/Entire Volume, Including Marrow

Supplemental Figure 5: High Correlation of Midshaft Femur and Midshaft Tibia Cortical Thickness in the Ovariectomy Study

The effect of genotype is far greater than the effect of ovariectomy (not shown).

Supplemental Figure 6: Correlations of Femur 4-Point Bending Strength (Maximum Load) with Cortical Thickness, Total Area and Polar Moment of Inertia in the Ovariectomy Study

For clarity, values from OVX and control bones within each genotype are combined. The Y-intercept for the cortical thickness correlation (minus 0.7) is close to zero. The slope of 0.14 indicates femur strength increases by 1.4 newtons for each 10 µm increment in cortical thickness.

Supplemental Figure 7: Effects of *Sfpr4* KO and Zoledronic Acid (ZA) Treatment on Cortical Bone Parameters

Male and female mice were examined at 68 and 24 weeks of age, respectively. Values are means ± SEM for 5 to 8 mice per group, with statistical analysis by two-factor ANOVA.

Supplemental Figure 8: Upregulation of *Sfrp4* Expression in during BMP2 Induced Differentiation of Murine C2C12 Myoblasts into Osteoblasts

`

The EMBL-EBI Expression Atlas (1) was employed to search for relevant published mouse *Sfrp4* gene expression data. This search identified a dataset providing expression data (GeneChip™ Mouse Genome 430 2.0 Array for 45,100 probes) during BMP2-induced differentiation of murine C2C12 myoblasts into osteoblasts (2). Probe “1451031_at” provides *Sfrp4* expression values. BMP2 induction of osteoblast differentiation was performed in both control C2C12 cells and C2C12 cells stably transfected with microRNA-378. Full experimental details are provided in the original publication (2).

1. Petryszak R, Keays M, Tang YA, Fonseca NA, Barrera E, Burdett T, et al. Expression Atlas update--an integrated database of gene and protein expression in humans, animals and plants. Nucleic Acids Res. 2016; 44:D746-D752.

2. Hupkes M, Sotoca AM, Hendriks JM, van Zoelen EJ, Dechering KJ. MicroRNA miR-378 promotes BMP2-induced osteogenic differentiation of mesenchymal progenitor cells. BMC Mol Biol. 2014; 15:1.

Supplemental Figure 9: *Sfrp4* Gene Knockout Targeting Strategy and Southern Blot Analysis


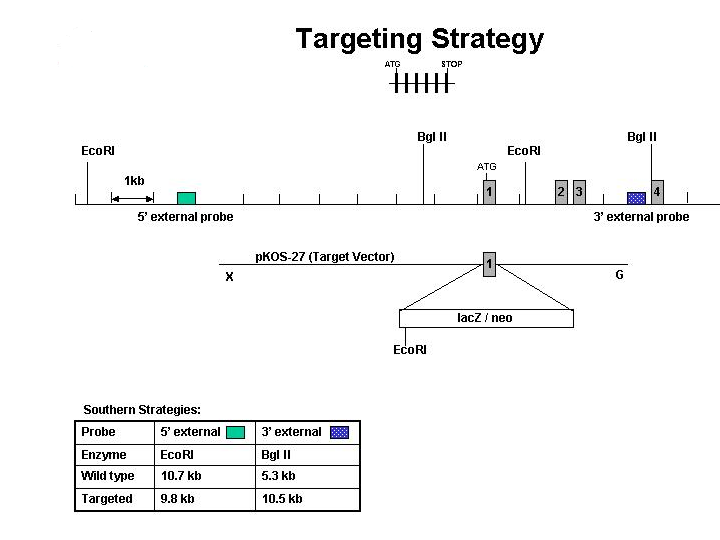


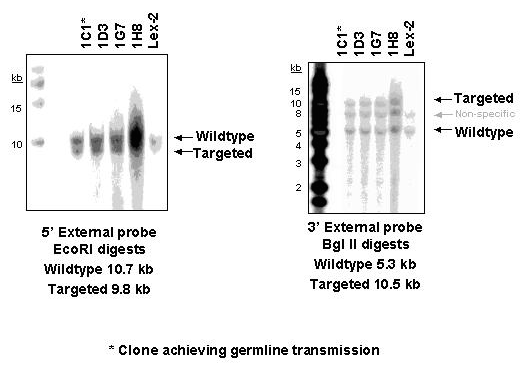


Cryopreserved sperm from this *Sfrp4* KO line are available from Taconic Biosciences, catalogue number TF3259 (<http://www.taconic.com/knockout-mouse/sfrp4-targeted>).

Supplemental Figure 10: Visualization of Spine LV5 Vertebral Body Cortical and Trabecular Bone Measurements


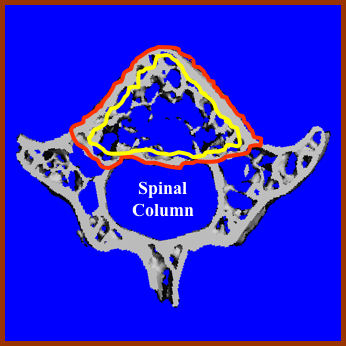


Trabecular bone is located inside the yellow contour line and cortical bone is located between the red and yellow contour lines. BV/TV values (with TV defined as the entire region inside the red line) for cortical and trabecular regions of the vertebral body are similar in control bones. This observation agrees with human spine distributions measured by dissection (Nottestad). Separate studies showed LV5 cortical bone mass, measured by this technique, is elevated with teriparatide treatment and in *Notum* KO mice (Brommage).

Manual contouring of the cortical-trabecular boundary was performed without knowledge of mouse genotypes.

The cortical shell contributes to vertebral compressive strength (Brommage, Christiansen, Vesterby).

Brommage R, Liu J, Vogel P, Mseeh F, Thompson AY, Potter DG, Shadoan MK, Hansen GM, Jeter-Jones S, Cui J, Bright D, Bardenhagen JP, Doree DD, Movérare-Skrtic S, Nilsson KH, Henning P, Lerner UH, Ohlsson C, Sands AT, Tarver JE, Powell DR, Zambrowicz B, Liu Q. NOTUM inhibition increases endocortical bone formation and bone strength. Bone Res. 2019; 7:2.

Christiansen BA, Kopperdahl DL, Kiel DP, Keaveny TM, Bouxsein ML. Mechanical contributions of the cortical and trabecular compartments contribute to differences in age-related changes in vertebral body strength in men and women assessed by QCT-based finite element analysis. J Bone Miner Res. 2011; 26):974-983.

Nottestad SY, Baumel JJ, Kimmel DB, Recker RR, Heaney RP. The proportion of trabecular bone in human vertebrae. J Bone Miner Res. 1987; 2:221-229.

Vesterby A, Mosekilde L, Gundersen HJ, Melsen F, Mosekilde L, Holme K, Sørensen S. Biologically meaningful determinants of the in vitro strength of lumbar vertebrae. Bone. 1991; 12:219-224.

Supplemental Table 1: *Sfrp4* KO Mouse Cohorts

Cohort 1: High-Throughput Screen with DEXA and microCT data at 16 - 17 weeks of age; see Brommage et al.; 2014.

Cohort 2: Male mice examined by microCT data at 7 weeks of age; OVX study with female mice

from 16 through 24 weeks of age with DEXA, microCT and bone strength analyses.

Cohort 3: Female mice examined by microCT data at 4 weeks of age; male mice examined with DEXA and microCT at 60 weeks of age.

Cohort 4: Aging mouse study; female mice examined at 92 weeks and male mice at 105 weeks.

Cohort 5: Male and female mice treated with zoledronate at 16 weeks of age; female mice examined with microCT at 28 weeks and male mice examined with DEXA and microCT at 68 weeks.

Cohort 6: *Sfrp4/Wnt16* DKO study examining males and females with microCT analyses at 16 weeks of age; see Brommage et al.; 2014.

Cohort 7: *Sfrp4/Notum* DKO study examining males and females with microCT analyses at 16 weeks of age. See Brommage et al., 2019.

Brommage R, Liu J, Hansen GM, Kirkpatrick LL, Potter DG, Sands AT, et al. High-throughput screening of mouse gene knockouts identifies established and novel skeletal phenotypes. Bone Res. 2014; 2:14034.

Brommage R, Liu J, Vogel P, Mseeh F, Thompson AY, Potter DG, Shadoan MK, Hansen GM, Jeter-Jones S, Cui J, Bright D, Bardenhagen JP, Doree DD, Movérare-Skrtic S, Nilsson KH, Henning P, Lerner UH, Ohlsson C, Sands AT, Tarver JE, Powell DR, Zambrowicz B, Liu Q. NOTUM inhibition increases endocortical bone formation and bone strength. Bone Res. 2019; 7:2.

Supplemental Table 2: Body Composition of *Sfrp4* KO Mice

Sex Cohort Age N Weight Lean Mass Fat Mass Fat Percent

(weeks) (grams) (grams) (grams) (%)

Female WT 2 16 20 24.1 ± 0.9 18.5 ± 0.5 5.5 ± 0.5 22.4 ± 1.1

Female KO 2 16 20 24.6 ± 0.7 19.2 ± 0.4 5.4 ± 0.4 21.5 ± 1.0

P = 0.63 P = 0.27 P = 0.84 P = 0.56

Male WT 5 16 17 23.1 ± 0.6 19.8 ± 0.6 3.3 ± 0.1 14.5 ± 0.5

Male KO 5 16 15 22.3 ± 0.6 23.6 ± 0.6 3.7 ± 0.2 15.8 ± 0.6

P = 0.81 P = 0.52 P = 0.07 P = 0.09

Female WT 5 16 11 19.3 ± 0.6 16.4 ± 0.4 2.9 ± 0.2 14.8 ± 0.5

Female KO 5 16 15 18.0 ± 0.3 15.3 ± 0.3 2.7 ± 0.1 14.9 ± 0.6

P = 0.03 P = 0.03 P = 0.29 P = 0.99

Male WT 3 60 11 44.7 ± 1.5 31.4 ± 0.7 13.3 ± 1.0 29.1 ± 1.7

Male KO 3 60 10 40.2 ± 1.9 30.1 ± 0.8 10.1 ±1.3 24.5 ± 2.1

P = 0.07 P = 0.24 P = 0.06 P = 0.09

Male WT 4 52 18 46.7 ± 1.0 29.8 ± 0.8 16.9 ± 1.0 35.9 ± 1.7

Male KO 4 52 13 42.0 ± 1.8 27.7 ± 0.7 14.2 ± 1.4 32.9 ± 2.2

P = 0.02 P = 0.09 P = 0.12 P = 0.29

Female WT 4 52 14 38.1 ± 2.4 21.3 ± 0.8 16.8 ± 1.9 42.3 ± 2.7

Female KO 4 52 20 35.4 ± 1.9 20.5 ± 0.7 14.9 ± 1.6 39.6 ± 2.7

P = 0.38 P = 0.45 P = 0.45 P = 0.48

Male WT 5 68 15 39.9 ± 2.1 27.1 ± 1.1 12.8 ± 1.1 31.1 ± 1.6

Male KO 5 68 12 37.1 ± 2.2 26.2 ± 1.0 10.8 ± 1.4 27.7 ± 2.1

P = 0.39 P = 0.59 P = 0.31 P = 0.24

Male WT 4 78 17 46.7 ± 1.0 29.8 ± 0.8 16.9 ± 1.0 35.9 ± 1.7

Male KO 4 78 11 42.0 ± 1.8 27.7 ± 0.7 14.2 ± 1.4 32.9 ± 2.2

P = 0.02 P = 0.09 P = 0.12 P = 0.29

Female WT 4 78 11 38.1 ± 2.4 21.3 ± 0.8 16.8 ± 1.9 42.3 ± 2.7

Female KO 4 78 16 35.4 ± 1.9 20.5 ± 0.7 14.9 ± 1.6 39.6 ± 2.7

P = 0.38 P = 0.45 P = 0.45 P = 0.48

Body composition was determined by DXA scans. Male mice at 68 weeks were part of the zoledronic acid study. Since zoledronic acid treatment did not affect body composition (P > 0.3), values for treated and control mice were combined. Data are means ± SEM for the number of mice indicted, with statistical analysis by Student’s t-test.

Supplemental Table 3: Clinical Chemistry Data for *Sfrp4* KO Mice at 2 Years of Age

Parameter Units Male WT Male *Sfrp4* KO Female WT Female *Sfrp4* KO

N - 11 8 9 14

Albumin g/dl 3.3 ± 0.1 3.1 ± 0.2 3.2 ± 0.1 3.2 ± 0.1

ALP units/l 61 ± 9 82 ± 20 110 ± 16 175 ±

ALT units/l 78 ± 24 97 ± 40 52 ± 9 51 ± 11

Bilirubin-T mg/dl 0.32 ± 0.04 0.41 ± 0.11 0.62 ± 0.12 0.54 ± 0.12

BUN mg/dl 19.6 ± 1.4 20.7 ± 2.09 21.8 ± 1.4 22.0 ± 0.8

Calcium mg/dl 9.7 ± 0.3 9.7 ± 0.2 9.9 ± 0.1 9.9 ± 0.1

Cholesterol mg/dl 153 ± 21 144 ± 29 138 ± 29 142 ± 13

Chloride mM 113 ± 1 113 ± 1 114 ± 1 112 ± 1

Creatinine mg/dl 0.094 ± 0.007 0.100 ± 0.006 0.130 ± 0.012 0.127 ± 0.011

Globulin g/dl 2.52 ± 0.16 2.78 ± 0.20 2.10 ± 0.18 2.42 ± 0.13

Glucose mg/dl 128 ± 6 120 ± 9 144 ± 8 142 ± 6

Potassium mM 5.7 ± 0.2 5.5 ± 0.1 5.3 ± 0.2 5.4 ± 0.2

Sodium mM 152 ± 1 151 ± 1 151 ± 1 151 ± 1

Phosphorus mg/dl 6.5 ± 0.3 6.0 ± 0.3 6.2 ± 0.3 6.1 ± 0.2

Total Protein g/dl 5.8 ± 0.2 5.8 ± 0.3 5.3 ± 0.2 5.7 ± 0.1

Triglycerides mg/dl 123 ± 12 94 ± 14 127 ± 13 98 ± 10

Uric Acid mg/dl 1.10 ± 0.17 1.64 ± 0.21 1.26 ± 0.16 1.32 ± 0.12

Values are means ± SEM for male and female mice at 105 and 92 weeks of age, respectively. Examining males and females separately for statistical differences by Student’s t-test, all P values > 0.05.

Supplemental Table 4: Complete Blood Cell Count Data for *Sfrp4* KO Mice at 2 Years of Age

Parameter Units Male WT Male *Sfrp4* KO Female WT Female *Sfrp4* KO

N - 10 7 9 13

RBCs 10^6^/μl 9.2 ± 0.7 9.0 ± 0.6 8.7 ± 0.6 9.7 ± 0.4

Hemoglobin gm/dl 13.1 ± 0.9 12.4 ± 0.8 14.0 ± 0.7 14.9 ± 0.5

WBCs 10^3^/μl 6.8 ± 0.4 7.3 ± 1.0 3.5 ± 0.6 4.2 ± 0.6

Neutrophils 10^3^/μl 1.9 ± 0.4 3.7 ± 1.0 1.1 ± 0.4 1.5 ± 0.2

Lymphocytes 10^3^/μl 4.1 ± 0.6 2.9 ± 0.7 1.6 ± 0.3 2.3 ± 0.4

Monocytes 10^3^/μl 0.59 ± 0.13 0.65 ± 0.21 0.33 ± 0.13 0.38 ± 0.08

Eosinophils 10^3^/μl 0.14 ± 0.04 0.15 ± 0.04 Many undetectable values

Platelets 10^3^/μl 1204 ± 107 1535 ± 152 599 ± 93 853 ± 56

(P < 0.03)

Values are means ± SEM for male and female mice at 105 and 92 weeks of age, respectively. Examining males and females separately for statistical differences by Student’s t-test, except for platelet counts inn female mice, all P values > 0.05.

Supplemental Table 5: Femur Lengths of *Sfrp4* KO Mice

Sex Age WT Length (mm) *Sfpr4* KO Length (mm) Statistics

Females 17 Weeks 15.8 ± 0.1 (12) 16.1 ± 0.1 (14) P = 0.04

Females 24 weeks 16.4 ± 0.2 (10) 16.6 ± 0.2 (10) P = 0.40

Males 60 Weeks 16.9 ± 0.2 (10) 16.9 ± 0.2 (10) P = 0.99

Values are means ± SEM for the number of mice indicted in parentheses. Statistical evaluation by Student’s t-test.

Supplemental Table 6: Whole Body, Spine and Femur DXA BMD Data for *Sfrp4* KO Mice

Sex Cohort Age N Body BMD Spine BMD Femur BMD

(weeks) (mg/cm2) (mg/cm2) (mg/cm2)

Female WT 2 16 20 51.1 ± 0.9 60.7 ± 1.9 73.7 ± 1.3

Female KO 2 16 20 49.6 ± 1.0 67.7 ± 1.9 69.1 ± 1.6

P = 0.28 P < 0.02 P < 0.04

Male WT 5 16 11 46.8 ± 0.6 58.5 ± 1.5 63.4 ± 1.7

Male KO 5 16 15 43.3 ± 0.5 62.6 ± 1.6 61.0 ± 2.0

P < 0.001 P = 0.08 P = 0.38

Female WT 5 16 15 47.2 ± 0.9 64.9 ± 2.3 59.4 ± 1.2

Female KO 5 16 11 46.2 ± 0.8 61.2 ± 1.2 52.3 ± 1.2

P = 0.39 P = 0.13 P < 0.001

Male WT 4 52 18 56.8 ± 0.9 53.2 ± 2.2 89.1 ± 2.4

Male KO 4 52 13 56.4 ± 1.1 65.4 ± 2.1 84.3 ± 2.8

P = 0.76 P < 0.001 P = 0.20

Female WT 4 52 14 56.0 ± 1.1 62.8 ± 2.5 81.5 ± 2.1

Female KO 4 52 20 55.7 ± 0.8 74.3 ± 1.9 76.3 ± 1.9

P = 0.85 P < 0.001 P = 0.07

Male WT 3 60 11 55.0 ± 0.9 52.0 ± 3.2 85.3 ± 2.2

Male KO 3 60 10 54.7 ± 1.1 63.7 ± 2.0 79.2 ± 2.1

P = 0.88 P = 0.007 P = 0.06

Male WT 4 78 17 52.7 ± 0.9 49.5 ± 1.7 77.2 ± 2.2

Male KO 4 78 11 49.3 ± 1.4 67.6 ± 2.8 74.6 ± 2.5

P = 0.07 P < 0.001 P = 0.46

Female WT 4 78 11 54.0 ± 1.6 49.3 ± 3.0 80.5 ± 2.0

Female KO 4 78 16 54.6 ± 1.3 70.9 ± 3.5 74.8 ± 2.6

P = 0.79 P < 0.001 P = 0.13

Data are means ± SEM for the numbers of mice indicated, with statistical evaluation by Student’s t-test.

Supplemental Table 7: Skeletal Parameters in Heterozygous and Homozygous *Sfrp4* KO Mice

Parameter Wild-Type Heterozygous Homozygous ANOVA

N 11 9 10

Spine DEXA BMD 52.0 ± 3.2 55.8 ± 3.5 63.7 ± 2.0 P = 0.03

(mg/cm^2^) (P = NS) (P = 0.03)

LV5 Trabecular 16.0 ± 0.9 21.0 ± 1.5 27.4 ± 1.4 P < 0.001

BV/TV (%) (P = 0.02) (P < 0.001)

LV5 Cortical 13.3 ± 0.6 14.9 ± 0.8 11.0 ± 0.9 P = 0.004

BV/TV (%) (P = 0.24) (P = 0.05)

Midshaft Femur 2.14 ± 0.8 2.14 ± 0.8 2.76 ± 1.1 P < 0.001

Total Area (mm^2^) (P = 0.99) (P < 0.001)

Midshaft Femur 1.16 ± 0.05 1.09 ± 0.03 0.83 ± 0.04 P < 0.001

Bone Area (mm^2^) (P = 0.64) (P < 0.001)

Midshaft Femur 216 ± 10 191 ± 10 117 ± 7 P < 0.001

Cortical Thickness (µm) (P = 0.09) (P < 0.001)

Midshaft Femur 0.70 ± 0.05 0.66 ± 0.04 0.71 ± 0.05 P = 0.74

Polar MOI (mm4)

Distal Femur 4.3 ± 0.6 6.5 ± 1.1 12.5 ± 1.3 P < 0.001

Trabecular BV/TV (%) (P = 0.26) (P < 0.001)

Distal Femur 2.03 ± 0.15 2.57 ± 0.22 3.91 ± 0.12 P < 0.001

Trabecular N (1/N) (P = 0.05) (P < 0.001)

Distal Femur 50.5 ± 2.0 49.6 ± 1.8 41.5 ± 1.8 P = 0.004

Trabecular Th (µm) (P = 0.93) (P = 0.004)

Distal Femur 1.55 ± 0.07 1.60 ± 0.08 3.07 ± 0.15 P < 0.001

Total Area (mm^2^) (P = 0.94) (P < 0.001)

Tibia-Fibula Junction 1.89 ± 0.07 1.85 ± 0.06 2.39 ± 0.10 P < 0.001

Total Area (mm^2^) (P = 0.39) (P < 0.001)

Tibia-Fibula Junction 1.12 ± 0.05 1.04 ± 0.02 0.82 ± 0.4 P < 0.001

Bone Area (mm^2^) (P = 0.92) (P < 0.001)

Tibia-Fibula Junction 266 ± 4 256 ± 5 205 ± 7 P < 0.001

Cortical Thickness (µm) (P = 0.39) (P < 0.001)

Proximal Tibia 9.3 ± 1.1 10.0 ± 1.4 17.9 ± 1.5 P < 0.001

Trabecular BV/TV (%) (P = 0.90) (P < 0.001)

Proximal Tibia 2.72 ± 0.15 3.08 ± 0.23 4.37 ± 0.16 P < 0.001

Trabecular N (1/N) (P = 0.03) (P < 0.001)

Proximal Tibia 58.2 ± 2.4 49.9 ± 2.1 48.2 ± 1.8 P = 0.005

Trabecular Th (µm) (P = 0.03) (P = 0.004)

Proximal Tibia 1.23 ± 0.04 1.28 ± 0.06 2.81 ± 0.11 P < 0.001

Total Area (mm^2^) (P = 0.92) P < 0.001

Male mice from Cohort 3 were examined at 60 weeks of age. For the distal femur and proximal tibia microCT scans, the scan lengths were 3.0 and 2.0 mm, respectively, starting at the growth plate. Data are means ± SEM with statistical analysis by one-way ANOVA, followed by Dunnett’s test (parentheses) for multiple comparisons to bones from WT mice. NS = nonsignificant.

Supplemental Table 8: Serum levels of PINP and CTX in the Zoledronic Acid Study

Parameter Units WT WT + Zoledronate KO KO + Zoledronate

N - 8 9 7/6 8

PINP ng/ml 29.0 ± 3.6 17.2 ± 1.5 31.5 ± 4.9 18.3 ± 1.8

CTX gm/dl 7.64 ± 0.83 4.27 ± 0.30 8.47 ± 1.33 5.25 ± 0.59

Data are means + SEM. Statistical evaluation by two/factor ANOVA

For PINP, P = 0.57 for effect of genotype, P < 0.001 for effect of treatment, P = 0.82 for interaction.

For CTX, P = 0.25 for effect of genotype, P < 0.001 for effect of treatment, P = 0.92 for interaction.

PINP is a marker for bone formation and CTX is a marker for bone resorption.
